# FDG-PET/CT imaging parameters for predicting spontaneous regression of methotrexate-associated lymphoproliferative disorder in patients with rheumatoid arthritis

**DOI:** 10.1038/s41598-022-19727-y

**Published:** 2022-09-13

**Authors:** Tomohiro Kameda, Shusaku Nakashima, Katsuya Mitamura, Yuka Yamamoto, Takashi Norikane, Hiromi Shimada, Risa Wakiya, Mikiya Kato, Taichi Miyagi, Koichi Sugihara, Rina Mino, Mao Mizusaki, Norimitsu Kadowaki, Hiroaki Dobashi

**Affiliations:** 1 Department of Internal Medicine, Division of Hematology, Rheumatology and Respiratory Medicine, Faculty of Medicine, Kagawa University, 1750-1 Ikenobe, Miki-cho, Kita-gun, Kagawa, 761-0793 Japan; 2 Department of Radiology, Faculty of Medicine, Kagawa University, 1750-1 Ikenobe, Miki-cho, Kita-gun, Kagawa, 761-0793 Japan

**Keywords:** Oncology, Rheumatology, Risk factors

## Abstract

In this study, we investigated the usefulness of FDG-PET/CT for predicting spontaneous regression in methotrexate-associated lymphoproliferative disorder (MTX-LPD). Twenty patients with rheumatoid arthritis who were diagnosed with MTX-LPD were enrolled in the study. These patients were divided into those who showed spontaneous regression (SR group: ten patients) and those who received chemotherapy after discontinuation of MTX (CTx group: ten patients). Between-group differences in potential biomarkers were compared, including clinical markers at the onset of LPD [serum LDH and interleukin 2 receptor (sIL-2R)], change in absolute number of peripheral lymphocytes (ΔALC) over follow-up, and the FDG-PET/CT-derived parameters of maximum standardized uptake value (SUVmax), mean SUV (SUVmean), peak SUV (SUVpeak), sum of the metabolic tumor volume (MTVsum), and sum of total lesion glycolysis (TLGsum). The levels of sIL-2R, MTVsum, and TLGsum were significantly lower in the SR group than in the CTx group. In addition, ΔALC was higher in the SR group. In conclusion, MTV and TLG values measured by FDG-PET/CT may be suitable for use as predictors of SR in patients with MTX-LPD.

## Introduction

Rheumatoid arthritis (RA) is one of the most common autoimmune inflammatory diseases. It is associated not only with synovitis, but also with various organ complications such as lung and heart lesions. Malignancies are the most important complications affecting the prognosis and life expectancy. It is reported that RA is associated with several types of malignancies, including lymphoproliferative disorders (LPDs). In addition, LPDs, including malignant lymphoma, are known to occur in RA patients treated with disease modifying antirheumatic drugs (DMARDs). In particular, LPD associated with methotrexate (MTX)-treated RA is often referred to as MTX-associated LPD (MTX-LPD). MTX-LPD can spontaneously regress after discontinuation of MTX, which makes it difficult to decide on treatment induction, especially the indication for chemotherapy. To date, changes in absolute lymphocyte count (ALC) after MTX discontinuation^1^ and serum soluble interleukin-2 receptor (sIL-2R) level at the time of diagnosis^2^ have been reported to be indicators of spontaneous regression of LPD, but are insufficient alone.

^18^F-Fluorodeoxyglucose positron emission tomography/computed tomography (FDG-PET/CT) is a useful modality for the diagnosis and staging of malignant tumors, including malignant lymphoma. In FDG-PET/CT, the standardized uptake value (SUV) is frequently used to evaluate tumor activity. In recent years, two other 3D FDG parameters, metabolic tumor volume (MTV) and total lesion glycolysis (TLG), have emerged as imaging parameters for the diagnosis and prognosis of various solid tumors, including lung and esophageal cancers. In this study, we investigated the usefulness of changes in ALC (ΔALC) and several serum biomarkers, including sIL-2R, as predictors of spontaneous regression of MTX-LPD after MTX discontinuation. In addition, we analyzed whether SUV, MTV, and TLG measured on FDG-PET/CT could predict the spontaneous regression of MTX-LPD.

## Results

**Figure 1:**
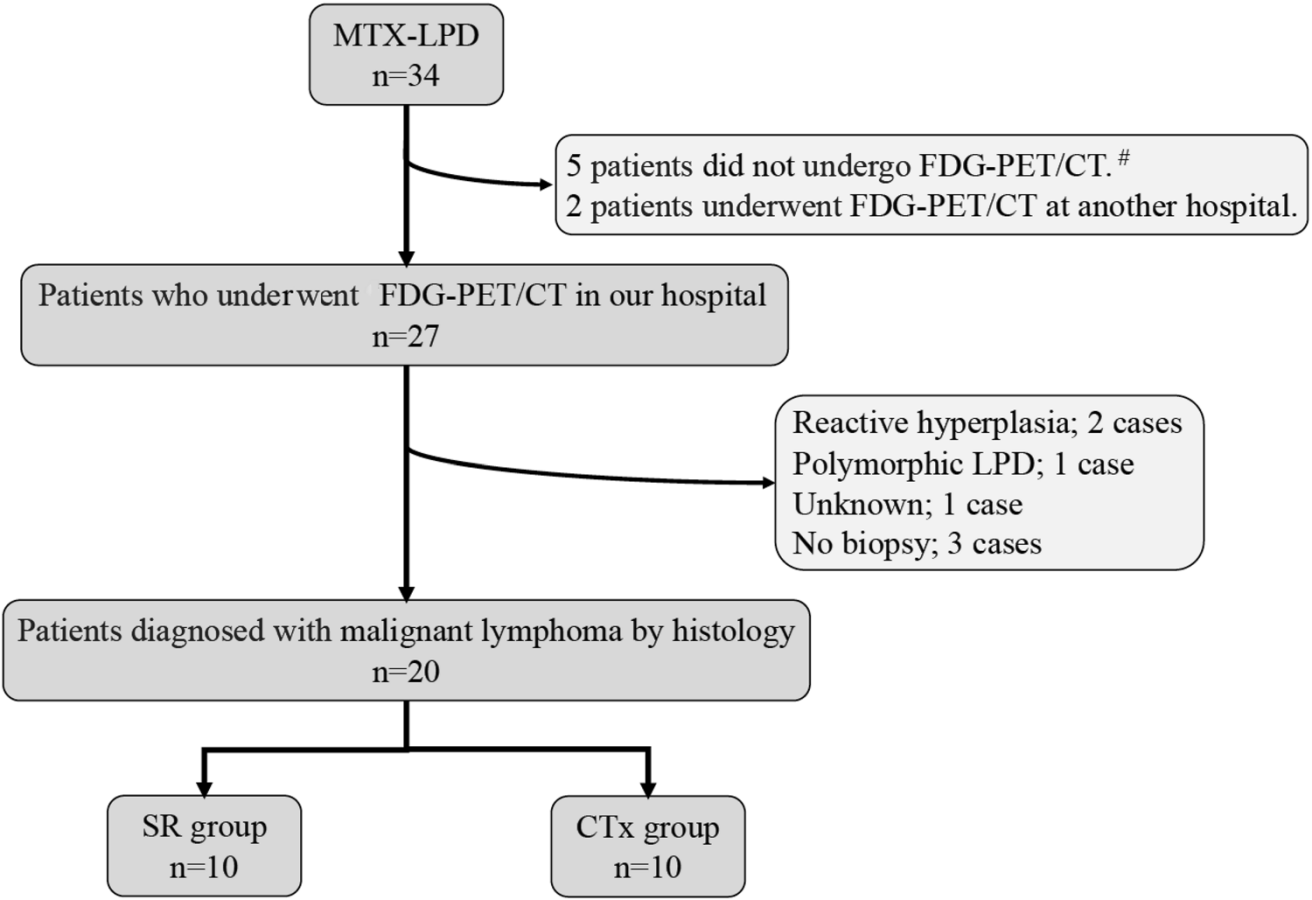
Patient selection flow chart. *SR* spontaneous regression, *CTx* chemotherapy. Hash: tumor rapidly regressed after discontinuation of MTX in four cases, whereas rapid growth occurred in one case.

Among 34 patients clinically diagnosed with MTX-LPD, 29 underwent FDG-PET/CT. Of the five patients who did not undergo FDG-PET/CT, four had rapid tumor regression after discontinuation of MTX and the other had rapid disease progression. Two cases underwent FDG-PET/CT at other institutions after being diagnosed with MTX-LPD and were excluded from this analysis. Of the 27 patients who underwent FDG-PET/CT at our institution, 20 were histopathologically diagnosed with malignant lymphoma. The remaining seven cases were histologically diagnosed as reactive hyperplasia (n = 2), pleomorphism (n = 1), unknown (n = 1), or were without tissue biopsy (n = 3), and were therefore excluded from the analysis. Finally, 20 cases diagnosed as malignant lymphoma were included in the analysis of this study (Fig. 1).

### Patient characteristics

The 20 MTX-LPD cases enrolled in the study were divided into a spontaneous regression (SR) group (n = 10) and a CTx group consisting of those who underwent chemotherapy after discontinuation of MTX (n = 10), as defined in the Methods. There were no differences in gender, age at onset of LPD, total MTX dose, mean MTX dose, or extranodal disease rate between the two groups, but the time from RA onset to LPD onset was shorter in the SR group. The mean disease activity over the year before LPD onset was no significant difference between the two groups.

The histological types of the malignant lymphomas were mostly diffuse large B-cell lymphoma (DLBCL) in the SR group and Hodgkin lymphoma (HL) in the CTx group (Table 1).

**Table 1:** Clinical profiles of the MTX-LPD patients.

		CTx (n = 10)	SR (n = 10)
Age at LPD onset (years)		62.5 [60, 70]	69.5 [66.5, 75.8]
Gender, M:F		2:8	2:8
Duration from onset of RA to LPD (years)		22 [18, 33]	9 [4, 12.8]
Disease activity of RA, DAS28-CRP		Remission: 6 LDA: 0 MDA: 2 HDA: 0 N.D.: 2	Remission: 3 LDA: 1 MDA: 3 HDA: 0 N.D.: 3
**MTX dose**			
Total (mg)		5948 [4597.5, 6387]	3418 [2288, 4312]
Mean (mg/week)		7.56 [6, 8.79]	8.46 [6.8, 10.8]
Extranodal involvement (%)		40.0	50.0
Histology		DLBCL: 1, HL: 6, IVL: 1, TCL: 1, MALT: 1	DLBCL: 6, HL: 1, FL: 1, MALT: 1, Unknown: 1

Median [IQR].

*LDA* low disease activity, *MDA* moderate disease activity, *HDA* high disease activity, *N.D.* no data, *DLBCL* diffuse large B-cell lymphoma, *HL* Hodgkin lymphoma, *IVL* intravascular lymphoma, *TCL* T cell lymphoma, *MALT* mucosa-associated lymphoid tissue, *FL* follicular lymphoma.

### Comparison of variables between the two groups

**Figure 2:**
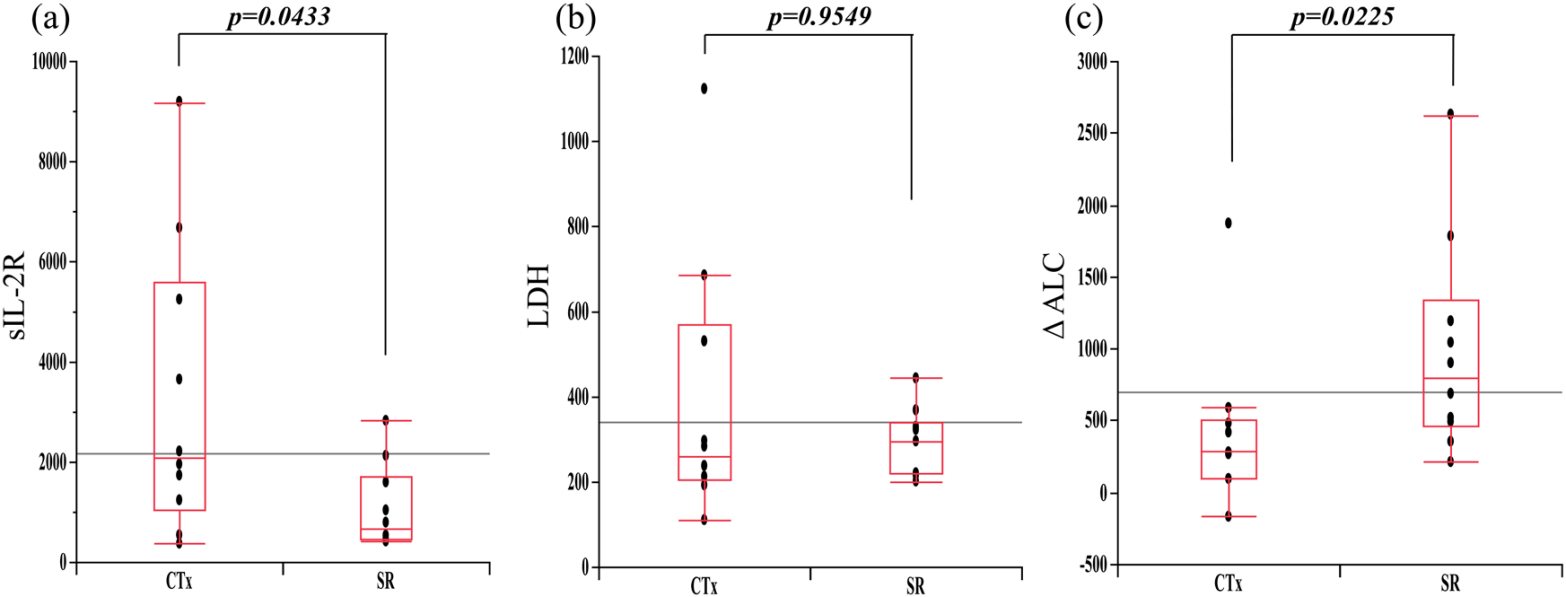
Comparisons of (**a**) sIL-2R (U/ml), (**b**) LDH (U/l), and (**c**) ΔALC (/µl) between SR and CTx groups.

**Figure 3:**
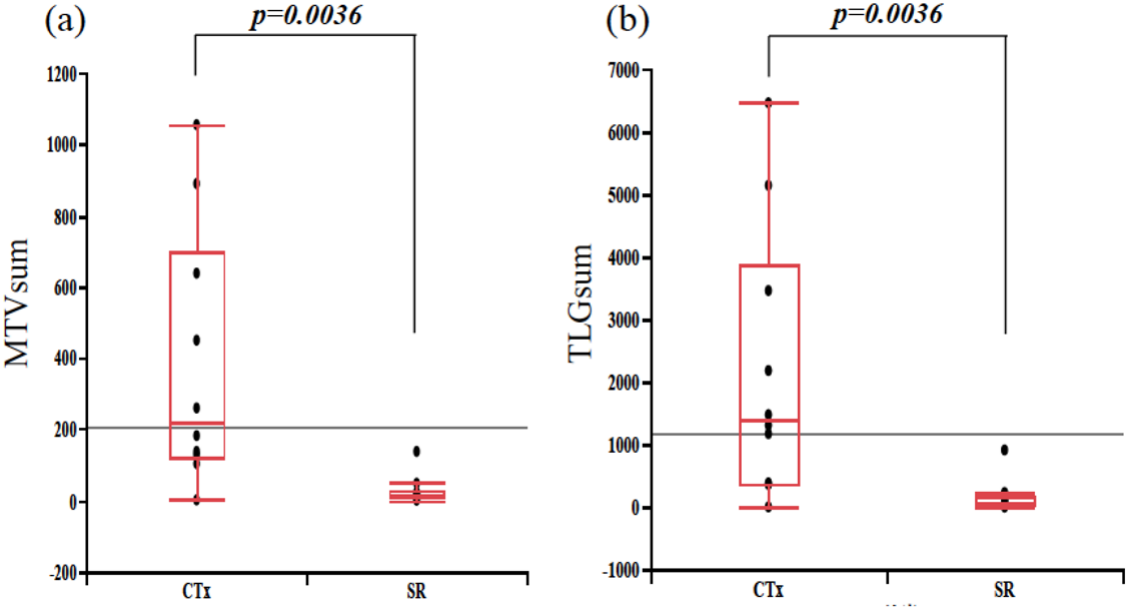
Comparisons of (**a**) MTVsum (ml) and (**b**) TLGsum between SR and CTx groups.

**Figure 4:**
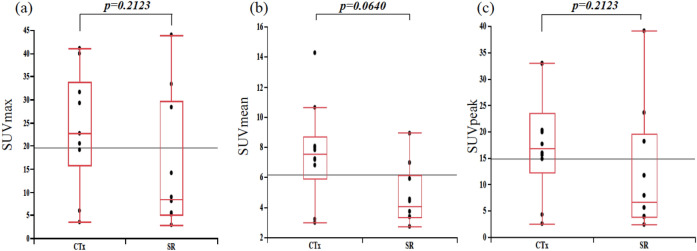
Comparisons of (**a**) SUVmax, (**b**) SUVmean, and (**c**) SUVpeak between SR and CTx groups.

The analysis of laboratory data at the onset of LPD showed no significant difference in LDH levels between the two groups, but sIL-2R was significantly lower in the SR group. In addition, ΔALC was significantly higher in the SR group than in the CTx group (Fig. 2). Analysis of imaging parameters obtained from FDG-PET/CT showed that MTVsum and TLGsum were significantly lower in the SR group (Fig. 3), but that SUVmax, SUVmean, and SUVpeak did not significantly differ between the two groups (Fig. 4).

## Discussion

This study is the first to report that MTV and TLG obtained from FDG-PET/CT imaging are useful for predicting spontaneous regression in patients with MTX-LPD. In 1991, Ellman reported for the first time that immunosuppressants such as MTX may be involved in the development of LPD in RA patients treated with them^3^. Subsequently, MTX-LPD, which develops in immunodeficient patients treated with MTX, was defined in the 2001 World Health Organization (WHO) Classification of Tumors of Haematopoietic and Lymphoid Tissues^4^. Nowadays, MTX-LPD is included in the “other iatrogenic immunodeficiency-associated LPD (OIIA-LPD)” category according to the 2017 WHO classification^5^.

MTX-LPD has the following characteristics^6^: (1) an age at onset of 60–70 years, (2) a mean duration from onset of RA to LPD of more than 10 years, (3) extranodal lesions in 40–70% of patients, and (4) spontaneous regression in 20–70% of patients after MTX cessation. In the 20 patients analyzed in this study, the age at LPD onset and the percentage of extranodal lesions were not significantly different between the two groups, but the mean duration from onset of RA to LPD was shorter in the SR group than in the CTx group. However, Kuramoto et al. reported that the time from RA onset to LPD onset was not related to spontaneous regression(SR)^7^. It is presumed that the duration of RA disease before LPD onset does not affect outcomes in the present study population as well.

At the time of diagnosis, it is difficult to determine whether or not a patient with MTX-LPD should be treated with interventional chemotherapy. Therefore, prediction of the spontaneous regression of LPD is important for rheumatologists and hematologists who treat MTX-LPD. Although Baecklund et al. reported that RA disease activity was a risk factor for LPD onset^8^, it is unknown whether the disease activity of RA influences the clinical course of LPD. Baecklund et al. scored RA disease activity at each visit for the whole RA disease period and defined the mean of the total scores from all visits as RA disease activity. In consideration of this, we defined the mean of the total DAS scores over the year before LPD onset as RA disease activity, and found that it was similar between the two groups. According to this result, we consider that the RA disease activity before LPD onset was of no relevance to the clinical course of LPD.

There have been several recent reports on predictors of spontaneous regression of LPD. Saito et al. reported that a decreased lymphocyte count at the time of LPD diagnosis and its restoration after MTX withdrawal were associated with spontaneous regression of LPD^1^, while Katsuyama reported that peripheral blood Epstein-Barr virus (EBV)-DNA positivity was a prognostic marker for MTX-LPD^9^. Tokuhira reported that sIL-2R is a useful biomarker for the management of MTX-LPD, including the decision on additional chemotherapy when LPD relapses after discontinuation of MTX^2^. In our study, the results of analyses of sIL-2R and ALC changes as predictors of spontaneous regression were consistent with these previous reports, but we attempted to further predict the prognosis of MTX-LPD using FDG-PET/CT imaging.

We investigated whether MTV and TLG calculated from FDG-PET/CT images acquired at the time of diagnosis can be used to predict the spontaneous regression of MTX-LPD. MTV and TLG are reported to be useful for predicting the prognosis of lung cancer^10,11^ and esophageal cancer^12,13^, and Luo et al. reported that MTV and TLG may predict the optimal timing of treatment for non-Hodgkin’s lymphoma^14^. However, various histological types of LPD are observed in MTX-LPD, and it is widely known that FDG uptake differs according to the histological type. In our study, most of the LPDs were DLBCL in the SR group and HL in the CTx group. However, when we compared the SUVmax, SUVmean, and SUVpeak of the LPDs, we found no significant differences in these imaging parameters between the histological types. Therefore, we consider that histological differences had little impact on the level of FDG uptake in MTX-LPD.

There have been several reports on the use of FDG-PET for prognostic prediction in patients with MTX-LPD. Watanabe et al. reported that no FDG-PET/CT parameters were useful for predicting complete response after discontinuation of MTX^15^, whereas Takanashi et al. reported that the SUVmax of FGD-PET/CT is a useful predictor of spontaneous regression^16^. However, there has been no study on the use of MTV and TLG PET imaging parameters for prediction of the spontaneous regression of MTX-LPD, which has diverse pathological and clinical features. In the present study, the MTV and TLG values at the time of MTX-LPD diagnosis were significantly lower in the SR group than in the CTx group. To the best of our knowledge, this is the first report of their use as predictive parameters for spontaneous regression in MTX-LPD.

In our analysis, SUVmax, SUVmean, and SUVpeak were not found to be predictive of spontaneous regression of MTX-LPD, whereas MTVsum and TLGsum could. Because the highest SUVmax, SUVmean, and SUVpeak among all the lesions showing FDG uptake were measured for each patient, these SUV parameters reflect a particular part of the MTX-LPD lesion. On the other hand, MTVsum and TLGsum quantify the entire lesion. The results of the present study suggest that FDG-PET/CT parameters assessing the entire lesion, namely MTVsum and TLGsum, are more useful than those assessing only part of the lesion, namely SUVmax, SUVmean, and SUVpeak.

In our results, the level of sIL-2R and the change in ALC are also predictive biomarkers of spontaneous regression in MTX-LPD. However, these biomarkers are affected by various factors such as the histological type of LPD and the disease activity of RA. Therefore, we consider the prognosis of LPD can be predicted more accurately by using the imaging parameters such as MTV and TLG combine with these biomarkers.

Our study has the following limitations: (1) it is a retrospective study; (2) the number of MTX-LPD cases analyzed is small; (3) we were not able to compare the two groups according to the presence or absence of EBV infection because of the small number of cases; (4) we did not investigate recurrence after SR; and 5) we did not apply a partial-volume-correction technique. Although partial-volume-correction is very important for measuring the true volume and accurately assessing metabolism, it is difficult to apply in daily clinical practice^17,18^.

In conclusion, our study revealed that MTV and TLG measured from FDG-PET/CT can be used as predictors of spontaneous regression in patients with MTX-LPD. We believe that these imaging parameters will be useful tools for the prompt determination of treatment strategy when MTX-LPD is diagnosed. In the future, it will be necessary to establish a more accurate method for predicting spontaneous regression by comprehensively evaluating multiple biomarkers and imaging parameters.

## Methods

### Participants and procedures

RA patients at Kagawa University Hospital who were diagnosed with RA according to the 1987 ACR or 2010 ACR/EULAR RA classification criteria, who were treated with MTX, and who developed MTX-LPD between January 2011 and July 2020 were selected. Among the RA patients identified as being diagnosed with MTX-LPD, those who underwent FDG-PET/CT imaging at our institution and pathological diagnosis by lesion biopsy were divided into a spontaneous regression group (SR group) and a chemotherapy group (CTx group) for analysis. The CTx group was defined as patients who required chemotherapy after discontinuation of MTX for at least 4 weeks. Clinical data for the SR and CTx groups were collected from medical records, and included gender, time from RA onset to LPD onset, age at LPD onset, disease activity of RA, total MTX dose, mean MTX dose, presence of extranodal lesions, and histological findings. The disease activity of RA was assessed using the 28-joint count C reactive protein score (DAS28-CRP) measured on each visit made during the year up to the time MTX was discontinued because of LPD onset. The final disease activity score of each patient was calculated as the mean of the DAS scores from all visits.

This study was approved by the Ethics Committee of the Faculty of Medicine, Kagawa University (approval numbers: H29-118), and a waiver for the requirement for written informed consent was granted because of the retrospective observational study design. Although we did not obtain written informed consent from all patients, we disclosed information on our research, and all patients could refuse to participate in the research if they so wished. All research was performed in accordance with relevant guidelines and regulations.

### Comparisons of laboratory data

Serum sIL-2R and lactate dehydrogenase (LDH) measurements at the onset of LPD were collected from medical records and compared between the two groups. In addition, a peripheral ALC was made at the onset of LPD and 4 weeks after discontinuation of MTX. The change in ALC between these two time points was calculated as ΔALC using the following formula, and was compared between the two groups. ΔALC formula: *ΔALC* = *(ALC after 4 weeks of MTX discontinuation) − (ALC at the onset of LPD).*

### Comparison of imaging parameters

All imaging was performed using a hybrid PET/CT scanner (Biograph mCT, Siemens Medical Solutions USA Inc, Knoxville, TN, USA). Patients were instructed to fast for more than 5 h before FDG administration. FDG-PET/CT imaging was visually assessed by two board-certified nuclear medicine physicians working independently. Any difference of opinion was resolved by consensus. Visually abnormal FDG uptake seen in locations unaccounted for by the normal biodistribution of FDG was interpreted as lesion. A board-certified nuclear medicine physician performed the semiquantitative analyses. Lesion boundaries of voxels were determined using a fixed SUV threshold of ≥ 2.5, as used in a previous report^19^. Any FDG uptake by arthritic tissue was excluded. The maximum SUV (SUVmax), mean SUV (SUVmean), and peak SUV (SUVpeak; a 1.2-cm-diameter sphere positioned to maximize the mean value) were calculated. The highest SUVmax, SUVmean, and SUVpeak among all the lesions showing FDG uptake were evaluated for each patient. MTV was defined as the lesion volume where the SUV exceeded a threshold value^20^. TLG was calculated by multiplying the MTV by the SUVmean^21^. The sums of the MTV and TLG of all lesions within a patient were defined as MTVsum and TLGsum, respectively. These imaging parameters were calculated using an open-source software tool developed for the efficient measurement of tumor volume on FDG-PET/CT^22^.

### Statistical analysis

The laboratory data (sIL-2R, LDH, ΔALC) and the imaging parameters (SUVmax, SUVmean, SUVpeak, MTVsum, TLGsum) were compared between the SR and CTx groups using univariate analysis performed with JMP software, version 15.0 (SAS Institute, Cary NC, USA). Data were analyzed for statistical significance using the Wilcoxon signed-rank test. *P* values less than 0.05 were considered statistically significant.

## Data Availability

The data analyzed in this study can be provided on written request to the corresponding author.

## References

[1] Saito S, Kaneko Y, Yamaoka K, Tokuhira M, Takeuchi T (2017). Distinct patterns of lymphocyte count transition in lymphoproliferative disorder in patients with rheumatoid arthritis treated with methotrexate. Rheumatology (Oxford).

[2] Tokuhira M, Tanaka Y, Takahashi Y, Kimura Y, Tomikawa T, Anan T (2020). The clinical impact of absolute lymphocyte count in peripheral blood among patients with methotrexate - associated lymphoproliferative disorders. J. Clin. Exp. Hematop..

[3] Ellman MH, Hurwitz H, Thomas C, Kozloff M (1991). Lymphoma developing in a patient with rheumatoid arthritis taking low dose weekly methotrexate. J. Rheumatol..

[4] Harris NL, Swerdlow SH, Jaffe ES (2001). Methotrexate-associated lymphoproliferative disorders. Pathology and Genetics of Tumours of Haematopoietic and Lymphoid Tissues Lyon.

[5] Gaulard, P., Swerdlow, S.H., Harris, N.L., Sundstrdm, C., & Jaffe, E.S. Other iatrogenic immunodeficiency-associated lymphoproliferative disorders. in *WHO Classification of Tumours of Haematopoietic and Lymphoid Tissues* (Swerdlow, S.H., Campo, E., Harris, N.L. *et al*. eds). 4th edn. 462–444 (IARC, 2017).

[6] Harigai M (2018). Lymphoproliferative disorders in patients with rheumatoid arthritis in the era of widespread use of methotrexate: A review of the literature and current perspective. Mod. Rheumatol..

[7] Kuramoto N, Saito S, Fujii T, Kaneko Y, Saito R, Tanaka M (2022). Characteristics of rheumatoid arthritis with immunodeficiency-associated lymphoproliferative disorders to regress spontaneously by the withdrawal of methotrexate and their clinical course: A retrospective, multicenter, case-control study. Mod. Rheumatol..

[8] Baecklund E, Sundström C, Ekbom A, Catrina AI, Biberfeld P, Feltelius N (2003). Lymphoma subtypes in patients with rheumatoid arthritis: Increased proportion of diffuse large B cell lymphoma. Arthritis Rheum..

[9] Katsuyama T, Sada KE, Yan M, Zeggar S, Hiramatsu S, Miyawaki Y (2017). Prognostic factors of methotrexate-associated lymphoproliferative disorders associated with rheumatoid arthritis and plausible application of biological agents. Mod. Rheumatol..

[10] Takahashi N, Yamamoto T, Matsushita H, Sugawara T, Kubozono M, Umezawa R (2016). Metabolic tumor volume on FDG-PET/CT is a possible prognostic factor for Stage I lung cancer patients treated with stereotactic body radiation therapy: a retrospective clinical study. J. Radiat. Res..

[11] Wen W, Piao Y, Xu D, Li X (2021). Prognostic value of MTV and TLG of ^18^F-FDG PET in patients with stage I and II non-small-cell lung cancer: a meta-analysis. Contrast Media Mol. Imaging..

[12] Takahashi N, Umezawa R, Takanami K, Yamamoto T, Ishikawa Y, Kozumi M (2018). Whole-body total lesion glycolysis is an independent predictor in patients with esophageal cancer treated with definitive chemoradiotherapy. Radiother. Oncol..

[13] Tustumi F, Duarte PS, Albenda DG, Takeda FR, Sallum RAA, Junior UR (2021). Prognostic value of 18F-fluorodeoxyglucose PET/computed tomography metabolic parameters measured in the primary tumor and suspicious lymph nodes before neoadjuvant therapy in patients with esophageal carcinoma. Nucl. Med. Commun..

[14] Luo Y, Zhang Y, Pan Q, Zhang Y, Li F (2020). 18F-FDG PET/computed tomography may predict the outcome of newly diagnosed indolent non-Hodgkin lymphoma in patients managed with initial 'watch-and-wait' approach. Nucl. Med. Commun..

[15] Watanabe S, Manabe O, Hirata K, Oyama-Manabe N, Hattori N, Kikuchi Y (2016). The usefulness of (18)F-FDG PET/CT for assessing methotrexate-associated lymphoproliferative disorder (MTX-LPD). BMC Cancer.

[16] Takanashi S, Nakazato T, Aisa Y, Ito C, Arakaki H, Osada Y (2018). The prognostic value of positron emission tomography/computed tomography in rheumatoid arthritis patients with methotrexate-associated lymphoproliferative disorders. Ann. Hematol..

[17] Alavi A, Werner TJ, Høilund-Carlsen PF, Zaidi H (2018). Correction for partial volume effect is a must, not a luxury, to fully exploit the potential of quantitative PET imaging in clinical oncology. Mol. Imaging Biol..

[18] Høilund-Carlsen PF, Edenbrandt L, Alavi A (2019). Global disease score (GDS) is the name of the game!. Eur. J. Nucl. Med. Mol. Imaging..

[19] Nakatsuka Y, Handa T, Nakamoto Y, Nobashi T, Yoshihuji H, Tanizawa K (2015). Total lesion glycolysis as an IgG4-related disease activity marker. Mod. Rheumatol..

[20] Biehl KJ, Kong FM, Dehdashti F, Jin JY, Mutic S, El Naqa I (2006). 18F-FDG PET definition of gross tumor volume for radiotherapy of non-small cell lung cancer: is a single standardized uptake value threshold approach appropriate?. J. Nucl. Med..

[21] Larson SM, Erdi Y, Akhurst T, Mazumdar M, Macapinlac HA, Finn RD (1999). Tumor treatment response based on visual and quantitative changes in global tumor glycolysis using PET-FDG imaging: The visual response score and the change in total lesion glycolysis. Clin. Positron Imaging.

[22] Hirata K, Kobayashi K, Wong KP, Manabe O, Surmak A, Tamaki N (2014). A semi-automated technique determining the liver standardized uptake value reference for tumor delineation in FDG PET-CT. PLoS ONE.

